# Granular Kidney and Vascular Changes

**Published:** 1898-03-12

**Authors:** 


### GRANULAR KIDNEY AND VASCULAR CHANGES.

The old question as to the relation between the changes in the kidney and those in the general vascular system in cases of so-called granular kidney cropped up again at a recent meeting of the Harveian Society. Dr. Samuel West read a paper on "Granular Kidney: and Why it is so Often Overlooked," in which he drew attention to the frequency with which the symptoms fail to point directly to the condition of the kidneys as the main disease. Especial emphasis was laid on hæmorrhage in various parts of the body as a symptom of granular kidney. Heart failure, pericarditis, dyspepsia, and skin eruptions, and various nervous symptoms were also pointed out as conditions often symptomatic of this disease, and it was stated that from a consideration of such cases as those mentioned it was obvious that it was only too easy to be misled by the prominent symptoms, and to miss the prime cause of the malady, namely, granular kidney. As a criticism on this paper Dr. Robert Maguire said that if the term "granular kidney" meant the actual condition of the kidney, his answer to Dr. West was that granular kidney was often overlooked because it was not present—that is, that either the kidney was healthy, or, if diseased, its condition was not the cause of the symptoms observed. He quoted two cases showing that the general vascular changes might take place while there was as yet no change in the kidneys, and said that, if such things could occur without granular kidney, it was obvious that, in cases in which the state of the kidney was not normal, that abnormality could not be the cause of the symptoms. This reminded the older members of times forty years ago. It was the old discussion over again. So does history repeat itself.

